# Antimicrobial Properties, Functional Characterisation and Application of *Fructobacillus fructosus* and *Lactiplantibacillus plantarum* Isolated from Artisanal Honey

**DOI:** 10.1007/s12602-022-09988-4

**Published:** 2022-09-29

**Authors:** Nicola De Simone, Maria Teresa Rocchetti, Barbara la Gatta, Giuseppe Spano, Djamel Drider, Vittorio Capozzi, Pasquale Russo, Daniela Fiocco

**Affiliations:** 1 Department of Agriculture Food Natural Science Engineering (DAFNE), University of Foggia, via Napoli 25, 71122 Foggia, Italy; 2 Department of Clinical and Experimental Medicine, University of Foggia, via Pinto 1, 71122 Foggia, Italy; 3 Institute of Sciences of Food Production, National Research Council (CNR) of Italy, c/o CS-DAT, Via Michele Protano, 71122 Foggia, Italy; 4 UMR Transfrontalière BioEcoAgro N° 1158, Univ. Lille, INRAE, Univ. Liège, UPJV, YNCREA, Univ. Artois, Univ. Littoral Côte d’Opale, ICV—Institut Charles Viollette, 59000 Lille, France

**Keywords:** Fructophilic lactic acid bacteria (FLAB), Probiotic, Biocontrol, Filamentous fungi, Immunomodulation, Table grape

## Abstract

Honey is a valuable reservoir of lactic acid bacteria (LAB) and, particularly, of fructophilic LAB (FLAB), a relatively novel subgroup of LAB whose functional potential for human and food application has yet to be explored. In this study, FLAB and LAB strains have been isolated from honeys of different floral origins and selected for their broad antimicrobial activity against typical foodborne pathogenic bacteria and spoilage filamentous fungi. The best candidates, two strains belonging to the species *Lactiplantibacillus plantarum* and *Fructobacillus fructosus*, were submitted to partial characterisation of their cell free supernatants (CFS) in order to identify the secreted metabolites with antimicrobial activity. Besides, these strains were examined to assess some major functional features, including in vitro tolerance to the oro-gastrointestinal conditions, potential cytotoxicity against HT-29 cells, adhesion to human enterocyte-like cells and capability to stimulate macrophages. Moreover, when the tested strains were applied on table grapes artificially contaminated with pathogenic bacteria or filamentous fungi, they showed a good ability to antagonise the growth of undesired microbes, as well as to survive on the fruit surface at a concentration that is recommended to develop a probiotic effect. In conclusion, both LAB and FLAB honey-isolated strains characterised in this work exhibit functional properties that validate their potential use as biocontrol agents and for the design of novel functional foods. We reported antimicrobial activity, cytotoxic evaluation, probiotic properties and direct food application of a *F. fructosus* strain, improving the knowledge of this species, in particular, and on FLAB, more generally.

**Supplementary Information:**

The online version contains supplementary material available at 10.1007/s12602-022-09988-4.

## Introduction

Lactic acid bacteria (LAB) are Gram-positive, non-sporulating, facultative anaerobic microorganisms, which are common on soil, plants, several types of food and within the gastro-intestinal tract of animals, including humans, where they are known to promote host gut functions [1]. LAB have been extensively studied, and their technological applications are vast and include *(i)* a consolidated use in food fermentation, where they enhance nutritional and organoleptic properties and enable bio-preservation, and *(ii)* the potential preparation of functional food and feed, as several LAB strains have been claimed probiotics [2]. Among LAB, the versatile and highly adaptable species of *Lactiplantibacillus plantarum* has a solid tradition of use in food and a more recent record of health claims and related applications as a probiotic [3]. LAB can exert antagonistic activity against other microbes through the competition for nutrients and by the production of different antimicrobials, including, among others, organic acids, bacteriocins, hydrogen peroxide, acetoin and fatty acids [4, 5]. This makes them attractive as protective cultures in food preservation, especially in view of eco-sustainable approaches [6]. For example, broad-spectrum antifungal LAB have been successfully applied in fruit models to protect against contamination by filamentous fungi [7], while *L. plantarum* has been reported as a promising agent to antagonise post-harvest fruit spoilage [8, 9] and for the biocontrol of moulds in different food matrices, including cereal-based beverages [10] and bread [11].

FLAB have been identified as a relatively novel subgroup of LAB, which prefer fructose as fermentable substrate and consequently prevail in fructose-rich habitats, such as flowers, fruits, honey and the gastro-intestinal tracts of insects feeding on them [12]. In honeybees gut, where FLAB seem to be the dominant bacterial species [13], such microbes contribute to metabolising sugars and contrasting pathogen growth, thus playing a relevant role in digestive functions and intestinal microbiota homoeostasis [14]. While the beneficial activity of symbiont FLAB is well acknowledged for insects such as honey bees [15–17], with a conspicuous body of research on the effect of their dietary supplementation on honey bee and beehive health [18], their probiotic and technological potential for human applications has yet to be explored [19].

Honey, a sweet fluid produced by honey bees and other insects from flower nectar, is a natural, high-energy food with distinctive functional properties (e.g. antioxidant, antimicrobial, antiviral, anti-inflammatory, antimutagenic and anticancer effects) that benefit human health [20]. Recently, honey was also proposed as a valuable reservoir of probiotics [21, 22]. So far, honey-isolated microbes with potential to enhance animal and human health comprise FLAB, such as *Lactobacillus kunkeei* [15, 23, 24] (recently reclassified as *Apilactobacillus kunkeei* [25]) and *Fructobacillus fructosus* [13, 15, 24], and members of the genus *Lactiplantibacillus*, *Lactobacillus* [24, 26], *Enterococcus* [27] and *Bacillus* [22].

The screening for microbial strains with probiotic potential can rely on well-established in vitro assays which help to evaluate some basic traits and to predict their efficacy in vivo [28]. For instance, tolerance to the gastrointestinal tract (GIT) conditions is a key criterion for selecting dietary probiotics, and GIT models, which simulate in vitro the different phases of human digestion, can be used to estimate whether the test microorganism can reach alive, and at effective doses, the host intestine [29, 30]. Another relevant characteristic of probiotics, i.e. their ability to colonise the host gut and persist on the mucosa, can be assayed in vitro by measuring microbial adhesiveness to mucus or to intestinal epithelial-like cells, such as Caco-2 or HT-29 [31, 32]. Likewise, immunomodulatory properties, which underlie most probiotic health benefits, can be preliminarily investigated in vitro by studying microbial interaction with host immune cells, such as macrophages or dendritic cells, thereby evaluating, for instance, the impact on cytokine production [33, 34].

In this work, LAB and FLAB strains were isolated from artisanal honey samples, subjected to molecular identification and characterised for functional and probiotic properties related to their possible utilisation as biocontrolling agents and as ingredients of health-promoting food, using table grape as a food model.

## Materials and Methods

### Honey Samples and Isolation of Presumptive LAB 

Three different honey samples, namely wildflower, coriander and orange honey, were collected from a local honey beekeeper in Foggia (Apulia, Italy). The samples were stored under refrigerated conditions before the analysis.

Honey (10 g) was aseptically resuspended in 90 mL of peptone water and homogenised using a stomacher. Thereafter, serial dilutions were spread onto de Man-Rogosa-Sharpe (MRS) (Oxoid, Basingstoke, UK) supplemented with CaCO_3_ (1.5 w/v) and incubated at 37 °C for 48 h in aerobic conditions. Presumptive LAB showing a clear zone around the colonies were isolated and cryoconserved at −80 °C in MRS containing 20% (v/v) of glycerol.

### Microbial Strains for Antagonistic Assays 

The filamentous fungi *Aspergillus niger* CECT 2805 and *Botrytis cinerea* CECT 20973 were purchased by the Colección Española de Cultivos Tipo (CECT, Paterna, Spain). Cryopreserved cultures were propagated on malt extract (Oxoid) agar plates at 24 °C for 5 days.

Three foodborne pathogenic bacterial strains, namely *Escherichia coli* O157:H7 UFG77, methicillin-resistant *Staphylococcus aureus* UFG141 and *Listeria monocytogenes* CECT 4031, were inoculated from cryopreserved stock (1:1000 v/v) in Tryptone Soya Broth (TSB) (Oxoid) and incubated at 37 °C for 24 h.

### Screening for Antimicrobial Activity

The antimicrobial activity of presumptive LAB isolates was determined against pathogenic bacteria and filamentous fungi using the agar overlay method, as reported by Russo et al. [10]. Briefly, 5 µL of LAB cultures at the late exponential phase were spotted on MRS agar plates and grown at 37 °C for 24 h. Then, plates were overlaid with 10 mL of TSB or malt extract soft agar (0.75% w/v of agar) containing the target bacteria or spores at a concentration of 10^6^ CFU (colony forming unit) mL^−1^, for antibacterial and antifungal activity, respectively. The zone of inhibition around the spot was measured after 48 h of incubation at 37 °C for bacterial pathogens and 3 days of incubation at 25 °C for filamentous fungi.

### Molecular Identification of the Investigated Isolates

Genomic DNA was extracted according to the method described by Singh and Ramesh [35]. Amplification of the 16S rRNA gene was carried out by using primer BSF8 (5´-AGAGTTTGATCCTGGCTCAG-3´) and BSR1541 (5´-AAGGAGGTGATCCAGCCGCA-3´). PCR was performed in a 20-µL reaction volume containing 20 ng of DNA, 10 µM dNTP mix, 0.2 nM of each primer and 2.5 U μL^−1^ Taq polymerase (Qiagen, Hilden, Germany). The PCR protocol was as follows: denaturation at 94 °C for 4 min, followed by 30 cycles at 94 °C for 30 s, 55 °C for 30 s, 72 °C for 90 s and a final extension at 72 °C for 5 min. Amplification products were checked by electrophoresis on a 1% agarose gel (w/v). PCR fragments were purified using the QIAquick PCR purification kit (Qiagen) and sequenced (Macrogen, Madrid, Spain). The strains were identified by homology search using Basic Local Alignment Search Tool (BLAST, http://www.ncbi.nlm.nih.gov/BLAST). The 16S rRNA sequences of the selected and investigated LAB isolates were deposited in the GenBank data library under accession numbers ON141890 (strain MEP3, *L. plantarum*) and ON141891 (strain AREP6, *F. fructosus*).

### Partial Characterisation of Antimicrobial Compounds in Cell-Free Supernatants (CFS)

Isolated strains of *L. plantarum* and *F. fructosus* with the highest and broadest antimicrobial activity, as determined by agar overlay method, were further investigated and submitted to a partial characterisation of the metabolites with antimicrobial activity according to Al Kassaa et al. [36]. *L. plantarum* and *F. fructosus* strains were grown for 24 h and 48 h, i.e. a time corresponding to late exponential and late stationary phase, according to previously generated standard growth curves. The corresponding supernatants were recovered by centrifugation (10,000 g × 5 min) and sterilised by filtration (0.45-μm filters, VWR international, West Chester, PA). Then, each CFS was aliquoted and submitted to different treatments: untreated; neutralised with 2 M NaOH (until pH 6.5); incubated with catalase (1 mg mL^−1^) (Sigma-Aldrich Corporation, USA) at 37 °C for 1 h.

### Antibacterial Activity of Cell-Free Supernatants (CFS) from Isolated Strains 

Pathogenic bacteria were grown in TSB and incubated at 37 °C for 24 h, then cultures were diluted (1:100 v/v) with TSB supplemented with 10%, 15% and 20% of sterile CFS from 48-h-old cultures of *L. plantarum* or *F. fructosus* isolated strains. The same assay was performed by using neutralised and catalase-treated CFS. Bacterial growth was monitored for 24 h at 37 °C in a BioTek Eon spectrophotometer (BioTek, Winooski, VT, USA), by determining each hour the OD_600_ after 5 s of shaking. The assays were carried out in triplicate.

### Antifungal Activity of Cell-Free Supernatants (CFS) from Isolated Strains

The antifungal activity of selected LAB and FLAB strains was further investigated by determining the radial growth inhibition of the hyphae, as described by Wang et al. [37]. CFS of LAB and FLAB strains grown for 24 h and 48 h was obtained as above reported. Then, each CFS was included in Potato Dextrose Agar (PDA) (Oxoid) at a concentration of 10% (v/v). The control was prepared using PDA containing 10% (v/v) of MRS. A culture containing 10^6^ spores mL^−1^ was spotted at the centre of the plate. Antifungal activity was determined by comparing the inhibition of radial growth of the hyphae to the control after 3 days of incubation at 24 °C. The same assay was performed by using neutralised and catalase-treated CFS. To evaluate the antifungal effect of different CFS concentrations, the supernatant obtained after 48 h of incubation was used to repeat this assay by supplementing PDA with increasing CFS levels, i.e. 15, 20% (v/v). All the assays were performed in triplicate.

### Quantification of Lactic Acid in the CFS and Antagonistic Assays

A growth curve was established by measuring viable cells and the amount of lactic acid. To this end, samples were withdrawn from *L. plantarum* or *F. fructosus* cultures at 0, 6, 24, 30 and 48 h of growth. Samples were centrifuged at 10,000 × g, for 10 min at 4 °C. Supernatants were filtered (0.2 μm), and then lactic acid was quantified by HPLC Spectra System P1000 XR (Thermo Electron Corporation, Madison, WI, US). The column used was a Fast Juice Column (50 mm × 7.8 mm, Phenomenex, Torrance, CA, US) with isocratic elution with H_3_PO_4_ (0.05% w/w), a flow rate of 0.8 mL min^−1^ and a temperature of 55 °C. The injected sample volume was 25 µL. A calibration curve was established by HPLC analysing increasing lactic acid (Sigma-Aldrich, St. Louis, MO, USA) concentrations from 1, 5, 7.5, 10 to 20 g L^−1^. The peak corresponding to lactic acid was identified using the Azur Software, which was eluted at a retention time of 5.32 min.

In order to quantitate the growth inhibitory effect of CFS caused by lactic acid, both antifungal and antibacterial assays were performed in the presence of the same percentages of MRS containing 18 g L^−1^ of lactic acid that was used as a negative control.

### HPLC Separation and Quantification of Other Organic Acids in the CFS

A liquid chromatograph Agilent 1100 Series system (Santa Clara, CA, USA) equipped with a Zorbax SB-C18 RRHT column (4.6 × 10 mm, 1.8 μm Agilent Technologies, Santa Clara, CA, USA) and a HPLC detector, UV-DAD, Agilent 1200 series was used to separate organic acids. Each sample (50 μL) was injected onto the column, and the separation was monitored at 214 nm for 30 min. The mobile phase was 0.1% (v/v) phosphoric acid in ultrapure water (HPLC grade) with a flow rate of 0.5 mL min^−1^. Tartaric acid, malic acid, ascorbic acid, acetic acid, citric acid, succinic acid and fumaric acid were used as standard, and the calibration curve was obtained from selected concentrations. The chromatographic peaks of the samples were identified according to the retention times of the standards. In the calculation of the organic acid amounts, the dilution of the samples is taken into account.

### In Vitro Tolerance to the Oro-Gastrointestinal Assay

Mid-exponential phase cultures of *L. plantarum* and *F. fructosus* (OD_600nm_ = 0.8) were harvested by centrifugation (8,000 g × 3 min) and resuspended into sterile saline solution (NaCl 8.6 g L^−1^) at a concentration of about 2 × 10^9^ CFU mL^−1^. The bacterial suspensions were subjected to a model mimicking the oro-gastrointestinal transit, as described by Bove et al. [38]. Briefly, oral stress (OG1) was simulated by adding 15 mg L^−1^ of lysozyme (Sigma-Aldrich) to a gastric electrolyte solution (6.2 g L^−1^ NaCl; 2.2 g L^−1^ KCl; 0.22 g L^−1^ CaCl_2_; 1.2 g L^−1^ NaHCO_3_) and incubating for 3 min at 37 °C. Then, 3 g L^−1^ of pepsin (Sigma-Aldrich) was added, and the samples acidified consecutively to pH 3.0 (OG2) and 2.0 (OG3), being each step incubated at 37 °C for 30 min. Subsequently, the intestinal environment was simulated by neutralising at pH 6.5 with 1 M NaOH and by adding 3 g L^−1^ of porcine bile salts and 1 g L^−1^ of pancreatin (all from Sigma-Aldrich), followed by incubation for 1 h at 37 °C (OG4). Finally, samples were diluted (1:1 v/v) with an intestinal electrolyte solution (5 g L^−1^ NaCl; 0.6 g L^−1^ KCl; 0.25 g L^−1^ CaCl_2_) to mimic the large intestine and incubated for 1 h at 37 °C (OG5). Samples from the different steps of the system were serially diluted and plated on MRS agar to determine viable cells. Survival to stress was determined relative to control unstressed samples. The assays were performed in triplicate.

### Adhesion to Human Intestinal Cell Lines

The human colorectal adenocarcinoma Caco-2 cell line was used to assess the in vitro adhesion assay. For this purpose, cells were grown in a controlled atmosphere of 5% CO_2_ at 37 °C in Dulbecco’s Modified Eagle’s Minimal Essential Medium (DMEM) (Biowest, Nuaillé, France), supplemented with 4.5 g L^−1^ glucose, 2 mM glutamine, 100 U mL^−1^ penicillin, 100 U mL^−1^ streptomycin, 1% (v/v) non-essential amino acids and 10% (v/v) fetal bovine serum (FBS). Monolayers of Caco-2 cells were grown in 24-well tissue culture plates by seeding 4 × 10^4^ cells per well and incubating for a week at 37 °C. Overnight *L. plantarum* or *F. fructosus* cultures were washed twice with PBS pH 7.4 (8000 × g, 10 min), resuspended in DMEM without antibiotics neither FBS and finally applied to confluent Caco-2 cell monolayers (10^6^ CFU per well). After 2 h of incubation at 37 °C (5% CO_2_ and 95% air), Caco-2 monolayers were washed twice with 500 mL of 1 × PBS to remove non-adherent bacteria and then detached by incubation at 37 °C for 15 min in the presence of trypsin/EDTA (Sigma). Then, samples were diluted and plated onto MRS agar to enumerate the adherent bacteria. CFU obtained from washed wells (cell-adhering bacteria only) were compared with those obtained by trypsinisation from control unwashed wells (total bacteria, i.e. adhering and non-adhering ones), in order to calculate the adhesion percentages [(CFU)_washed well_/(CFU)_unwashed well_] × 100.

### Cytotoxicity and Cell Viability Assays

HT-29 cells were cultured at 37 °C in a humid atmosphere with 5% CO2 in DMEM medium supplemented with 4.5 g L^−1^ glucose, 2 mM L-glutamine, 100 U mL^−1^ penicillin, 100 U mL^−1^ streptomycin, 1% (v/v) non-essential amino acids and 10% (v/v) FBS in 75 cm^3^ Falcon-type vials or in 96-well plates. For cytotoxicity assays, an inoculum of 8 × 10^3^ cells per well was prepared 1 week before the experiment. During this time, three changes of medium were carried out to maintain optimal conditions for the growth of HT-29 cells. The selected LAB and FLAB strains were grown for 36 h at 37 °C in MRS broth. The cultures were centrifuged (10 min, 8,000 × g, 4 °C) and resuspended in DMEM without any antibiotic or FBS to achieve a MOI 10:1, allowing the contact with HT-29 cells for 24 h at 37 °C and 5% CO_2_. The assays were carried out in triplicate, and triton 1% was used as negative control. To measure the cytotoxic effect, the cells were washed twice with PBS in order to eliminate any bacteria, then DMEM medium containing gentamicin (50 μg mL^−1^) and 5% of CCK-8 reagent ‘Cell Counting Kit-8’ (Dojindo Molecular Technology, Rockville, MD, USA) was added to quantify HT-29 cell viability. Living HT-29 cells were able to reduce tetrazolium salt by their dehydrogenase activity. After 2 h of incubation in the dark, the resulting orange-coloured product was quantified by measuring the absorbance at 450 nm with a microplate reader (Xenius, Safas, Monaco, Monaco). The data obtained were expressed as a percentage of survival of HT-29 cells compared to the control (i.e. HT-29 cells without contact with bacteria).

### Stimulation of Human Macrophages and Gene Expression Analysis 

Human monocytoid leukaemia-derived cells, THP-1 (from Sigma-Aldrich), were cultivated in RPMI-1640 (Gibco, Carlsbad, CA) supplemented with 10% (v/v) fetal bovine serum (FBS), 2 mM L-glutamine, 50 U mL^−1^ penicillin and 50 μg mL^−1^ streptomycin, in 5% CO_2_, at 37 °C. Immunostimulation experiments were performed as previously reported [39]. Briefly, THP-1 cells were seeded (5 × 10^5^ cells/well) in 24-well tissue culture-treated plates (EuroClone, Milan, Italy), and 100 ng mL^−1^ phorbol 12-myristate 13-acetate (PMA) (Sigma-Aldrich) was added to induce differentiation into macrophages. After 48 h, THP-1-derived macrophages were treated with 100 ng mL^−1^ of lipopolysaccharides (LPS) from *E. coli* O127:B8 (Sigma-Aldrich) and co-incubated with live bacterial cells from mid-exponential phase cultures (OD_600nm_ = 0.8) of strains MEP3 or AREP6 in a ratio of 1:1000 (macrophages: bacteria) according to Arena et al. [33] and based on preliminary MTT cytotoxicity test. After 3-h incubation, total RNA was isolated from macrophages using TRIzol reagent (Ambion, Thermo Fisher Scientific, Waltham, MA), checked by gel electrophoresis, quantified (BioTek Instruments, Winooski, VT) and reverse-transcribed using QuantiTect Reverse Transcription Kit (Qiagen, Valencia, CA). The relative expression level of immune-related genes was assessed by quantitative real-time RT-PCR (qRT-PCR), as previously described [32]. Negative and positive controls were unstimulated macrophages and macrophages stimulated only with LPS, respectively. Glyceraldehyde-3-phosphate dehydrogenase (GAPDH) and β-actin mRNA levels were used as internal controls to normalise the expression of target genes, according to the 2^−ΔΔCt^ method [40]. The oligonucleotides used for qRT-PCR are shown in Table S1 of supplementary material.

### Evaluation of MEP3 and AREP6 as Biocontrolling Agents and as Ingredients of Health-Promoting Food by Using Table Grape

Table grape var. *Italia* was obtained from a local farm and stored at 4 °C. The berries were manually separated from the rachis, sanitised by immersion in water containing HNaClO (100 ppm) for 30 s and air-dried. Cultures of bacterial pathogens (i.e. *E. coli* O157:H7 UFG77, *S. aureus* UFG141 and *L. monocytogenes* CECT 4031) at late exponential phase and fungal strains (i.e. *A. niger* CECT 2805 and *B. cinerea* CECT 20973) were obtained as above reported. *L. plantarum* MEP3 and *F. fructosus* AREP6 were grown in 1 L of MRS for 48 h at 37 °C, biomass recovered by centrifugation (5,000 × g for 3 min), washed two times and resuspended in 600 mL of sterile saline solution. The solution was aliquoted in six sterile plastic containers containing 100 mL. Then, fungal and pathogenic cultures were inoculated at a ratio of 1:100 (v/v) in the corresponding solutions of MEP3 and AREP6, and in physiological solution for control samples. Fifteen berries for each experimental condition were contaminated by immersion for 30 s, air-dried and packed in plastic cups, each containing one berry in passive-modified atmosphere packaging conditions. Assays were performed in triplicate. Bacterial viability was determined by plate counting on MRS, CEC, *Listeria* selective agar base and Mannitol Salt Agar (all from Oxoid) to enumerate LAB or FLAB, *E. coli*, *L. monocytogenes* and *S. aureus*, respectively. Fungal contamination was visually monitored. Analyses were carried out after 0, 3, 7, 10 and 14 days of storage at 4 °C.

### Statistical Analysis

One-way analysis of variance (ANOVA) followed by post hoc Tukey HSD test was used to analyse data and determine any statistically significant difference, with *p* < 0.05 as the minimal level of significance.

## Results

### Screening of Antimicrobial Activity of LAB Isolated from Honey

Presumptive LAB (Gram-positive, catalase-negative, producers of organic acids) were isolated on MRS supplemented with CaCO_3_. Wildflower and coriander honeys had a contamination of about 6 × 10^4^ CFU mL^−1^, while a lower microbial load (7 × 10^2^ CFU mL^−1^) was found in orange honey. A total of 43 colonies (namely, 18 colonies from wildflower and coriander honeys, and 7 from orange honey) were selected for further investigation.

Using the agar overlay assay, all the strains were screened for their antagonistic activity against three typical foodborne pathogenic bacteria, i.e. *E. coli* O157:H7, methicillin-resistant *S. aureus* and *L. monocytogenes*, and two spoilage filamentous fungi, i.e. *A. niger* and *B. cinerea*. The isolates were classified as no, mild or strong antagonistic strains based on the zone of inhibition around the spots (Supplementary Table S2). Seven strains showing the highest and broadest spectrum antimicrobial activity were identified by 16S rRNA sequencing as *L. plantarum* (two and three strains from honey wildflower and coriander, respectively) and *F. fructosus* (two strains from orange honey).

### Effect of Incubation Time and Concentration on the Antifungal Activity of CFS

The CFS of two representative strains for each species, i.e. *L. plantarum* CNP4 and MEP3 (from coriander and wildflower honey, respectively) and *F. fructosus* AREP2 and AREP6 (from orange honey), were obtained from late exponential and stationary phase cultures, checked for pH and tested for antifungal activity against *B. cinerea* CECT 20973 and *A. niger* CECT 2805 by measuring hyphal radial growth inhibition (Table 1). As shown, both *L. plantarum* strains were able to acidify the media faster than *F. fructosus*, resulting in a higher antifungal activity after 24 h. However, after 48 h of incubation, pH and antifungal activity were similar for the CFS from both species, resulting in higher acidity and enhanced inhibitory effect compared to those detected at 24 h.

**Table 1:** pH and antifungal activity of CFS obtained from cultures of *L. plantarum* (CNP4 and MEP3) and *F. fructosus* (AREP2 and AREP6) grown in MRS after 24 h and 48 h of incubation at 37 °C. Hyphal radial growth inhibition of *B. cinerea* CECT 20,973 and *A. niger* CECT 2805 was determined after 3 days of incubation at 24 °C in plates of PDA supplemented with 10% of MRS (control), or 24-h CFS and 48-h CFS. Results are expressed as the hyphal diameter (mm) and the corresponding percentage of inhibition. Mean values and standard deviation of three replicates are indicated

	**24-h CFS**			**48-h CFS**		
	pH	*B. cinerea* CECT 20973	CFS *A. niger* CECT 2805	pH	*B. cinerea* CECT 20,973	*A. niger* CECT 2805
Control	6.2	9.0 ± 0.0	4.2 ± 0.2	6.2	9.0 ± 0.0	4.2 ± 0.1
CNP4	3.8	6.4 ± 0.1 (28.9%)	3.6 ± 0.2 (14.8%)	3.6	5.9 ± 0.3 (34.4%)	3.5 ± 0.2 (16.7%)
MEP3	3.8	6.4 ± 0.2 (28.9%)	3.4 ± 0.2 (19.1%)	3.6	5.6 ± 0.2 (37.8%)	3.3 ± 0.1 (21.5%)
AREP2	5.7	9.0 ± 0.0 (0%)	4.2 ± 0.2 (0%)	3.6	6.1 ± 0.3 (32.2%)	3.4 ± 0.2 (19.0%)
AREP6	5.7	9.0 ± 0.0 (0%)	4.2 ± 0.3 (0%)	3.7	5.7 ± 0.2 (36.7%)	3.4 ± 0.0 (19.0%)

In order to ascertain the nature of the compounds responsible for antagonistic activity, the assay was performed by using neutralised and catalase-treated CFS. As shown in supplementary Fig. S1, the lack of inhibition under neutralised conditions and the persistence of inhibition after catalase treatment suggested that organic acids were actively involved in the antagonistic mechanisms. Interestingly, *B. cinerea* was more sensitive since its growth inhibition was almost twofold higher than that observed for *A. niger*. Since no significant differences were detected among the two strains of the same tested species, the effect of increasing concentrations of CFS taken after 48 h of incubation was investigated only for *L. plantarum* MEP3 and *F. fructosus* AREP6 (Table 2). The inhibition was higher by increasing the CFS concentration, and this pattern was more evident against *B. cinerea*. However, the addition of CFS obtained from *L. plantarum* MEP3 was always responsible for a greater inhibition than *F. fructosus* AREP6.

**Table 2:** Dose-depending antifungal activity of CFS. Hyphal radial growth inhibition was determined after 3 days of incubation at 24 °C in plates of PDA supplemented with 15 or 20% of MRS (control), or 48-h CFS from *L. plantarum* MEP3 and *F. fructosus* AREP6. Results are expressed as the hyphal diameter (mm) and the corresponding percentage of inhibition. Mean values and standard deviation of three replicates are indicated

	*B. cinerea* CECT 20973		*A. niger* CECT 2805	
	15%	20%	15%	20%
Control	9.0 ± 0.0	9.0 ± 0.0	4.2 ± 0.1	4.2 ± 0.2
MEP3	4.9 ± 0.3 (45.6%)	3.1 ± 0.2 (65.8%)	3.0 ± 0.3 (28.6%)	2.8 ± 0.2 (33.3%)
AREP6	4.9 ± 0.2 (45.6%)	4.2 ± 0.2 (53.2%)	3.2 ± 0.2 (23.8%)	3.0 ± 0.1 (28.6%)

### Effect of CFS Concentration on Antibacterial Activity

**Fig. 1:**
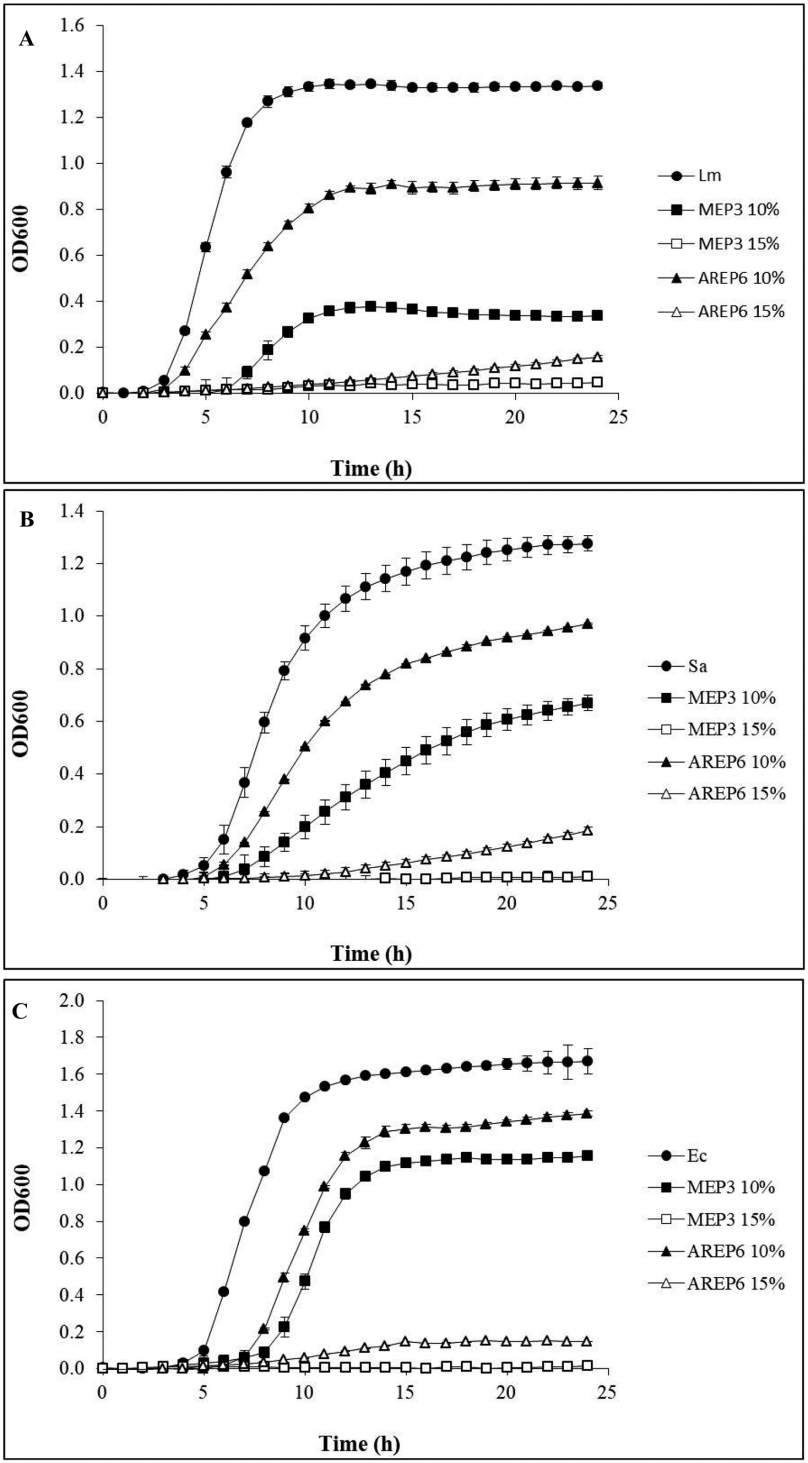
Kinetics of bacterial growth inhibition by CFS of *L. plantarum* MEP3 and *F. fructosus* AREP6. *L. monocytogenes* CECT 4031 (A), *S. aureus* UFG141 (B) and *E. coli* O157:H7 UFG77 (C) were inoculated in TSB supplemented with 10% of MRS (circle), or with 10% (black symbol), or 15% (white symbol) of 48 h-CFS obtained from *L. plantarum* MEP3 (square), or *F. fructosus* AREP6 (triangle). The cultures were incubated at 37 °C for 24 h, and optical density (OD_600_) was measured at 1-h intervals. Results are the average and SD of three assays

The antibacterial activity of the CFS from 48 h cultures of *F. fructosus* AREP6 or *L. plantarum* MEP3 was investigated by monitoring the pathogen’s growth in media supplemented with an increasing concentration of CFS. Figure 1 shows the growth kinetics of the indicator strains when 10% or 15% (v/v) of untreated CFS was added to the medium. In general, the addition of 10% CFS from either strains resulted in a reduction of the growth of all the pathogens, and the inhibitory effect was always more pronounced in the presence of MEP3’s CFS. Interestingly, an almost complete inhibition was detected in media supplemented with 15% of untreated CFSs. As expected, the growth of all pathogens was completely suppressed when 20% of untreated CFS was added (data not shown). No inhibition of growth was detected by adding neutralised CFS, while a similar inhibition than for untreated CFS was observed in the presence of catalase-treated CFS (Supplementary Fig. S2).

### Quantification of Lactic Acid in the CFS 

**Fig. 2:**
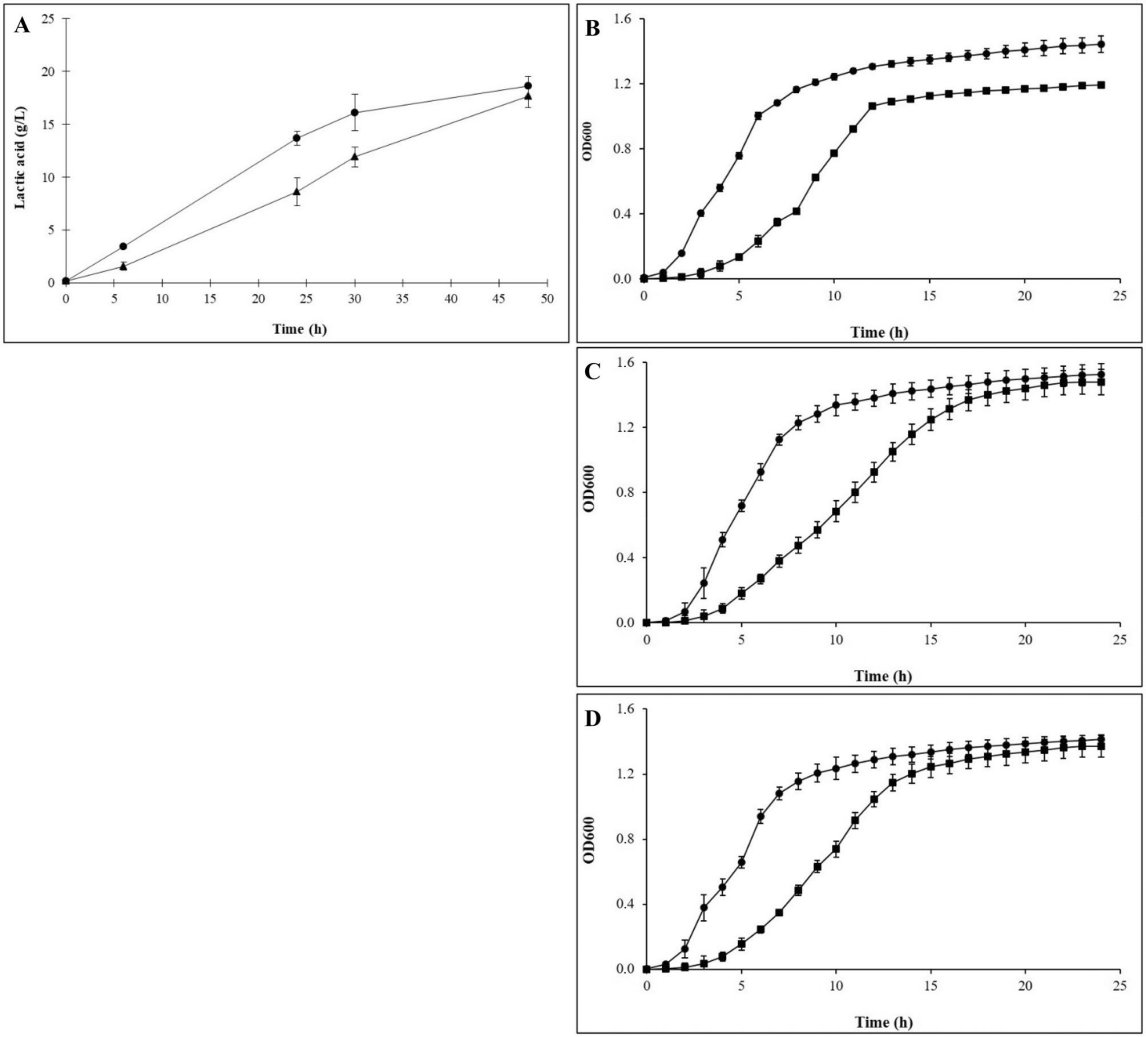
Quantification of the lactic acid produced by *L. plantarum* MEP3 (circle) and *F. fructosus* AREP6 (triangle) after 6, 24, 30 and 48 h of growth in MRS at 37 °C (**A**), and kinetics of bacterial growth of *L. monocytogenes* CECT 4031 (**B**), *S. aureus* UFG141 (**C**) and *E. coli* O157:H7 UFG77 (**D**) inoculated in TSB supplemented with 10% of MRS (circle), or with 10% of MRS containing 18 g L^−1^ of lactic acid (square). The cultures were incubated at 37 °C for 24 h, and optical density (OD600) was measured at 1-h intervals. Results are the average and SD of three assays

The amount of lactic acid produced by the two investigated honey-isolated strains was determined by HPLC of their CFSs. Figure 2A delineates the lactic acid production during the growth of both strains. In particular, the amount of lactic acid produced by strain MEP3 after 24 h of growth was 1.5-fold higher than AREP6, a result consistent with the lower pH observed for *L. plantarum* MEP3 at that cultivation phase. Nonetheless, after 48 h of growth, the level of lactic acid was similar and ranged from 18.6 and 17.7 g L^−1^ for MEP3 and AREP6, respectively.

In order to quantitate the growth inhibitory effect of CFS caused by lactic acid, the antimicrobial assays have been repeated in the presence of lactic acid, as negative control, at the concentration range corresponding to that obtained by HPLC. The addition of lactic acid inhibited the hyphal radial growth of *B. cinerea* CECT 20973 and *A. niger* CECT 2805 in a similar way than untreated CFS (Supplementary Fig. S3). Likewise, the addition of lactic acid negatively impacted the growth kinetics of the three target pathogens, albeit to a lesser extent than whole CFS (Fig. 2B–D). These results suggest that lactic acid is not the only compound responsible for the total growth inhibition detected in these experiments.

### Quantification of Other Organic Acids in the CFS

In order to determine the occurrence of other acid compounds with presumptive antimicrobial activity, some main organic acids were quantified by HPLC analysis of CFS (Table 3). In particular, both strains produced high concentrations of citric acid leading to an increase of this compound of two- and threefolds than in MRS for AREP6 and MEP3, respectively. In contrast, succinic acid and tartaric acid were more abundant in the CFS of AREP6, while only a little increase was detected for MEP3. A higher reduction of the content of malic acid in the CFS of MEP3 could suggest a better ability of *L. plantarum* than *F. fructosus* to perform the malolactic fermentation. No production of acetic, ascorbic and fumaric acids was found in both CFS.

**Table 3:** Main organic acids in MRS and 48-h CFS of *F. fructosus* AREP6 and *L. plantarum* MEP3 as determined by HPLC. Results (mg L^−1^) are the average and SD of three assays

Organic acid	MRS	AREP6	MEP3
Tartaric	503.27 ± 20.85	853.65 ± 73.57	692.22 ± 86.25
Malic	3,786.64 ± 95.81	2,543.97 ± 53.88	1,859.98 ± 636.10
Ascorbic	51.80 ± 1.30	n.d.*	n.d
Acetic	17,906.53 ± 162.88	18,144.59 ± 623.43	17,182.22 ± 143.82
Citric	1,206.78 ± 228.38	2,477.30 ± 158.10	3,356.90 ± 334.36
Succinic	1,066.96 ± 27.55	1,779.23 ± 144.00	1,208.37 ± 90.77
Fumaric	n.d	n.d	n.d

^*^n.d. not detected

### In Vitro Probiotic Characterisation of Honey-Isolated Strains

**Fig. 3:**
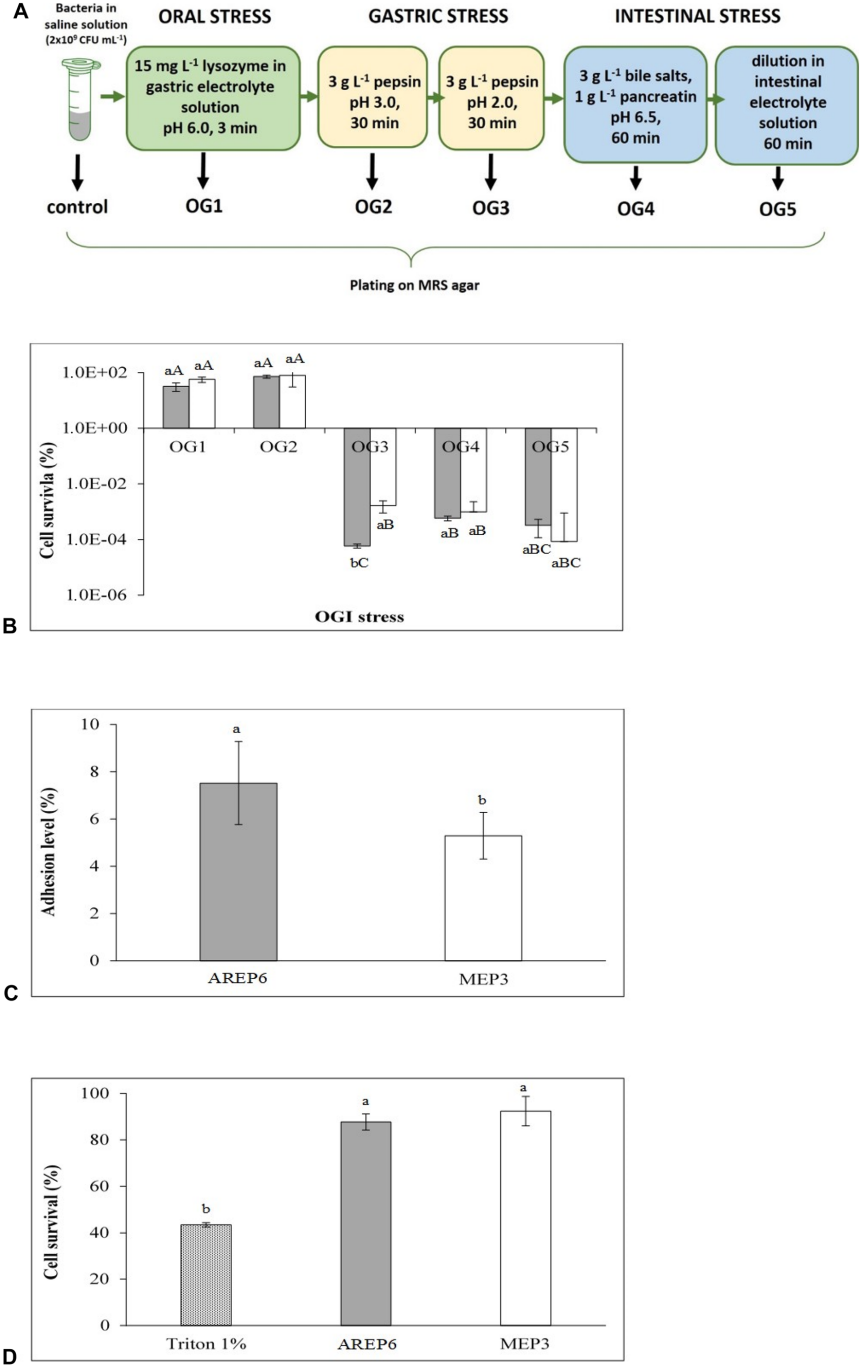
Probiotic characterisation of *L. plantarum* MEP3 and *F. fructosus* AREP6. Schematic diagram of the in vitro simulated oro-gastro-intestinal (OGI) transit (**A**) and relative survival of *F. fructosus* AREP6 (grey bars) and *L. plantarum* MEP3 (white bars) at its different steps **(B)**. Percentage of adhesion to Caco-2 monolayers normalised by using unwashed wells as control **(C)**. Survival of HT-29-cells upon 24-h contact with AREP6 and MEP3 **(D)**. Values are mean ± SD of three different experiments. Statistically significant differences were determined by one-way ANOVA and Tukey’s multiple comparison test (*P* < 0.05). Capital letters indicate significant differences among different steps of the OGI transit. Lowercase letters indicate significant differences between the strains

In order to perform a preliminary characterisation of their probiotic potential, *L. plantarum* MEP3 and *F. fructosus* AREP6 were investigated in vitro for their ability to face typical oro-gastrointestinal stress conditions by sequentially exposing the bacterial cultures to lysozyme, acidic pH and pepsin, pancreatic enzymes and bile salts, hence mimicking mouth, stomach and gut environments, respectively (Fig. 3A). As shown in Fig. 3B, no significant reduction of the viability was observed in the first two steps (i.e. lysozyme and pH 3). However, when the acidic conditions were further exacerbated (pH 2), a drastic decrease in survival was detected, corresponding to about 5 and 7 log units for *L. plantarum* MEP3 and *F. fructosus* AREP6, respectively, which indicated a different capability to challenge acidic stress between the two strains. Nonetheless, under simulated intestinal conditions (i.e. presence of bile and pancreatin at pH 6.5), survival seemed to be partially restored by *F. fructosus* AREP6, since the viable cells were similar to *L. plantarum* MEP3 (about 10^3^ CFU mL^−1^). However, both strains showed a slightly significant lower survival after prolonged exposure to simulated intestinal conditions (samples OG5).

In order to estimate the ability to colonise the gut mucosa, AREP6 and MEP3 strains were assayed for adhesion on intestinal epithelial-like cell monolayers. The scores of adhesion to human Caco-2 cells, performed after 2 h of incubation and 10:1 multiplicity of infection, ranged from 5.3 to 7.5%, with a significantly upmost percentage of adhesion for AREP6 (Fig. 3C). From a safety perspective, it was also useful to evaluate whether intestinal cells would retain viability upon interaction with the LAB. The tested strains were devoid of any cytotoxic effect against HT-29 cells. Indeed, HT-29 cells in contact with AREP6 and MEP3 presented a survival percentage of 87.7% and 92.4%, respectively, with no statistically significant difference between the tested microbial strains (Fig. 3D).

**Fig. 4:**
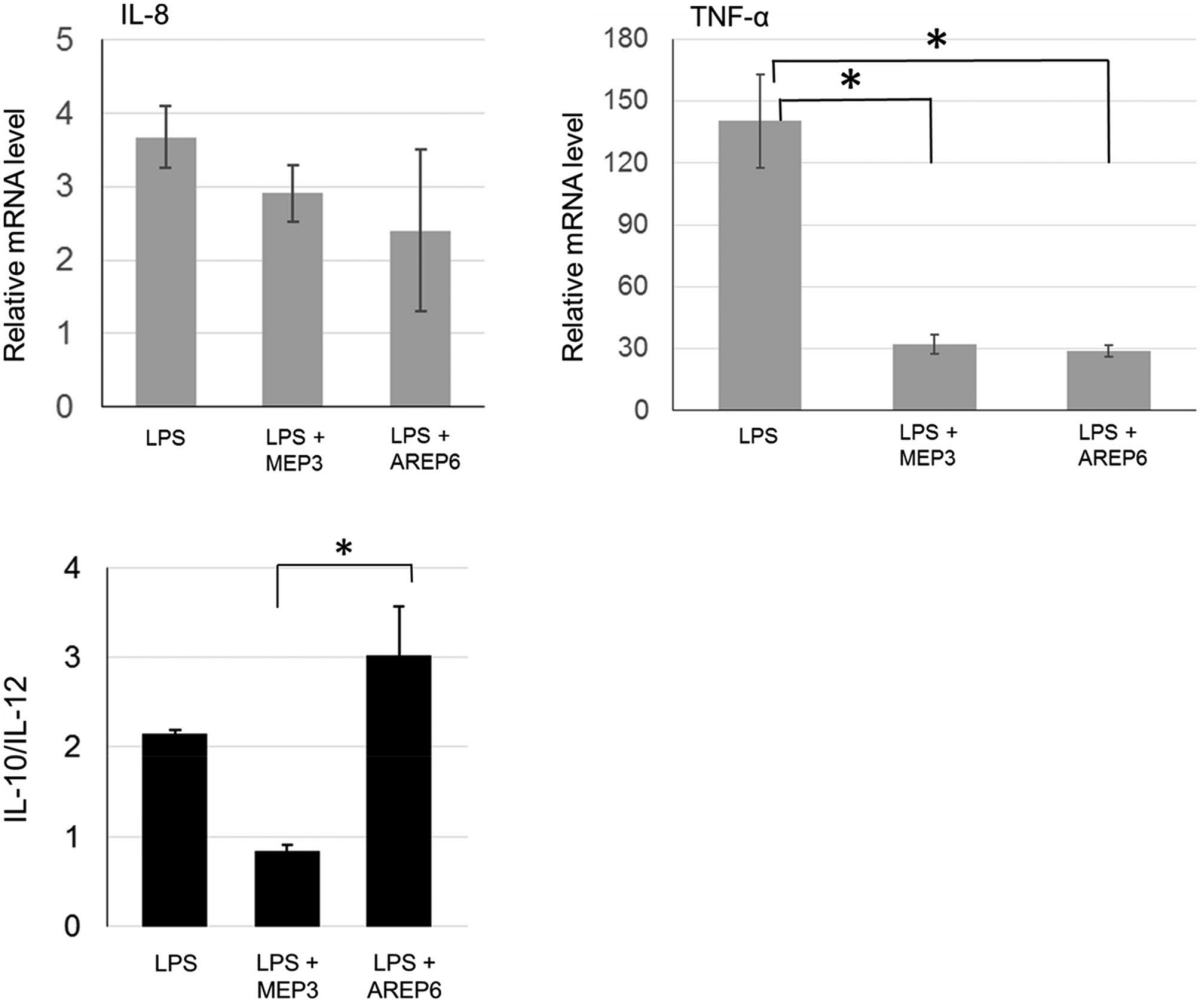
Immunomodulatory properties of *L. plantarum* MEP3 and *F. fructosus* AREP6. Relative transcriptional level of IL-8 and TNFα genes and IL-10/ IL-12 expression ratio. Relative mRNA levels were determined in LPS-stimulated macrophages with or without co-incubation with live bacterial cells from MEP3 or AREP6. Relative gene expression was determined by qRT-PCR after 3-h stimulation and was obtained by normalising to the level observed in non-LPS-stimulated macrophages (negative control, i.e. gene expression level set at 1). Mean ± SEM of at least two different experiments performed in triplicates. Statistically significant differences were assessed by one-way ANOVA (*p* value set at 0.05) and Tukey’s multiple comparison test: *, *p* ≤ 0.05

The capacity of MEP3 and AREP6 to modulate cytokine expression was evaluated in vitro on LPS-stimulated human macrophages (Fig. 4). As expected, the transcriptional level of the gene encoding pro-inflammatory cytokine IL-8 was upregulated by LPS, but slightly decreased when LPS was used in combination with cells from either strains. Likewise, TNF-α gene was strongly induced by LPS, but a consistent and statistically significant decrease in its transcriptional activation was observed when cells from either strains were co-incubated with LPS, thus indicating that these lactobacilli could attenuate pro-inflammatory stimulation. The transcriptional level of IL-10 and IL-12 was investigated since a high ratio between these two cytokines was previously correlated to the anti-inflammatory potential of candidate probiotic LAB [41, 42]. As shown in Fig. 4, in LPS-stimulated macrophages, the presence of *F. fructosus* AREP6 live cells determined a significantly higher IL-10/IL-12 mRNA ratio compared to *L. plantarum* MEP3.

### Evaluation of MEP3 and AREP6 as Biocontrolling Agents and Probiotics on Table Grape

**Fig. 5:**
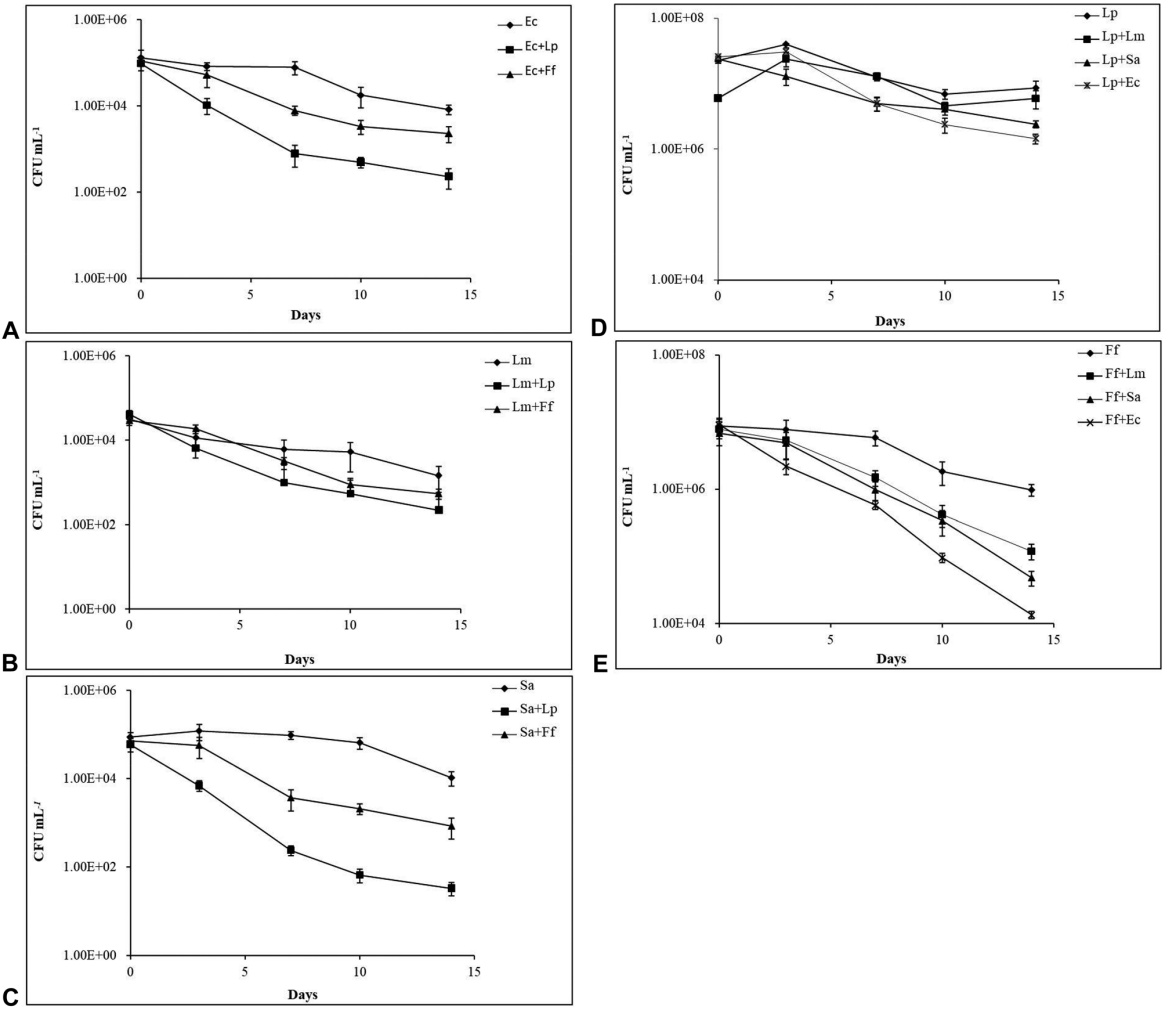
Viability of *E. coli* O157:H7 UFG77 (Ec) (**A**), *L. monocytogenes* CECT 4031 (Lm) (**B**), *S. aureus* UFG141 (Sa) (**C**) on table grapes when inoculated alone or with *L. plantarum* MEP3 (Lp) or *F. fructosus* AREP6 (Ff). Viability of *L. plantarum* MEP3 (**D**) and *F. fructosus* AREP6 (**E**) on table grapes when inoculated alone or with the indicated pathogens. Viability was monitored along 14 days of storage at 4 °C in passive-modified atmosphere. Mean and SD from three different experiments

Based on their antimicrobial and probiotic attributes, as determined by in vitro assays, *L. plantarum* MEP3 and *F. fructosus* AREP6 were tested further for their bioprotective and functional potential by using table grapes as a food model. Figure 5 shows the viability of three foodborne pathogenic bacteria, i.e. *E. coli* O157:H7 UFG77, *L. monocytogenes* CECT 4031 and *S. aureus* UFG141, whose growth was found to be inhibited in vitro by CFS from *L. plantarum* and *F. fructosus* (see above, Fig. 1), when they were inoculated on table grapes alone or together with strains MEP3 or AREP6. The initial contamination by the pathogens was about 1 × 10^5^ CFU g^−1^, a value that slightly and steadily decreased by about 1–2 log units during the cold storage of the berries. However, when grapes where artificially contaminated with both pathogens and each of the investigated LAB, *L. plantarum* MEP3 was always able to inhibit the viability of the pathogens to a higher extent than *F. fructosus* AREP6 (Fig. 5A–C). In particular, *S. aureus* was the most sensitive strain since its viability dropped to 3 × 10^1^ and 8 × 10^2^ CFU g^−1^ in the presence of MEP3 and AREP6, respectively (Fig. 5C). Although both LAB were inoculated at a level of approximately 1 × 10^7^ CFU g^−1^, only *L. plantarum* MEP3 was able to survive at high concentrations until the end of shelf life (8 × 10^6^ CFU g^−1^), while *F. fructosus* AREP6 showed a lower persistence on the surface of the grapes (about 1 log lower). Interestingly, in grapes contaminated also with the pathogens, the viability of MEP3 was only minimally reduced. In contrast, AREP6 viability was strongly affected, since its concentration fell down to 1 × 10^5^ and 1 × 10^4^ CFU g^−1^ in the presence of *L. monocytogenes* and *E. coli*, respectively (Fig. 5D, E), probably due to a higher sensitivity to competitive interactions with other microbial species.

**Fig. 6:**
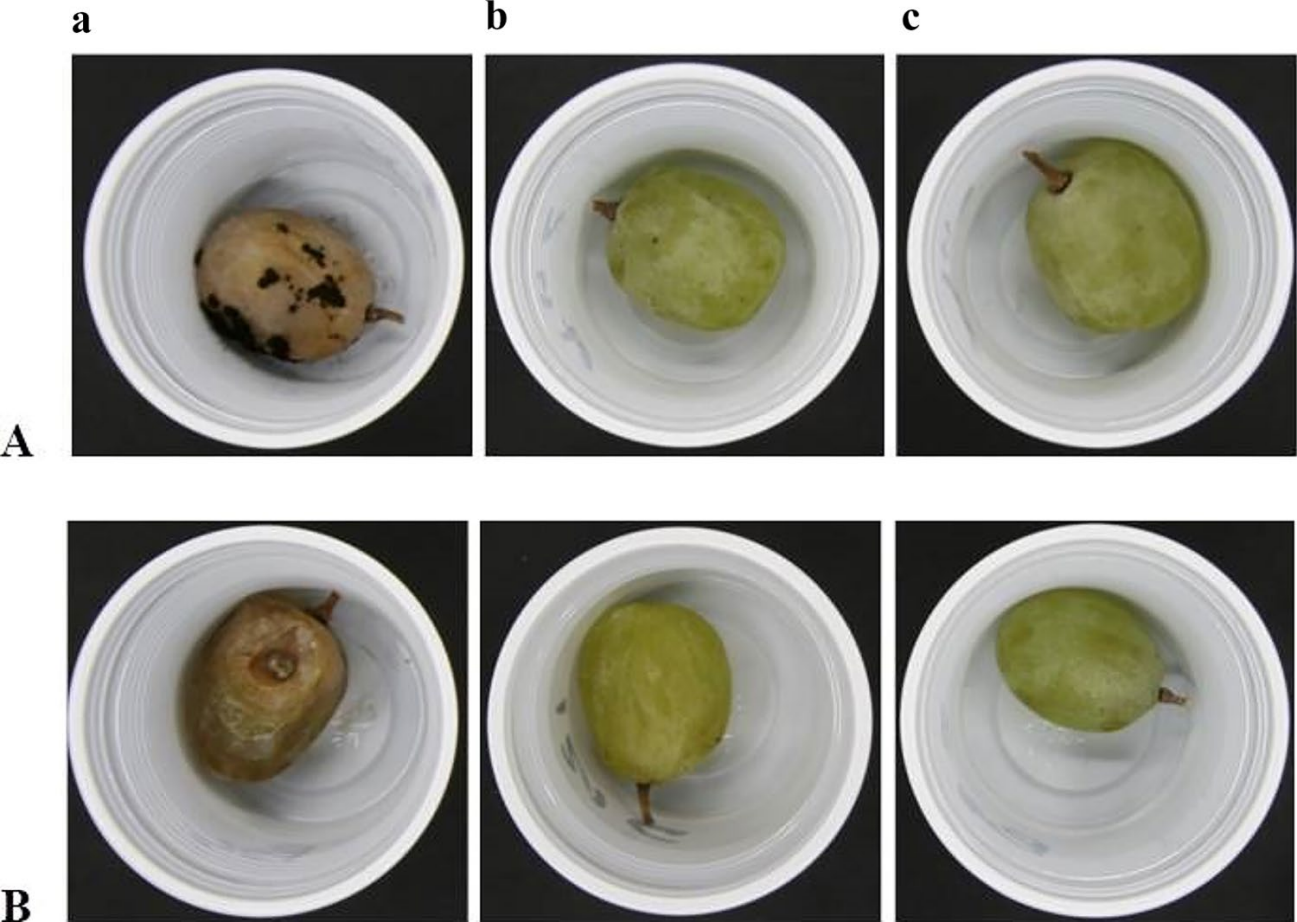
Table grapes artificially contaminated with *A. niger* CECT 2805 (**A**) or *B. cinerea* CECT 20,973 (**B**) without LAB (a) or in the presence of *F. fructosus* AREP6 (b) or *L. plantarum* MEP3 (c) after 14 days of storage at 4 °C

The ability of selected honey LAB strains to counteract the growth of moulds on the grapes was also investigated. The same fungal species, previously investigated in vitro and found to be sensitive to CFSs from the honey-isolated strains (see above, Tables 1 and 2), were used to contaminate table grapes, with or without addition of MEP3 or AREP6. As shown in Fig. 6, unlike control samples, i.e. grapes artificially contaminated with either *A. niger* CECT 2805 or *B. cinerea* CECT 20973, without LAB, the overall quality of the berries which were contaminated also with either LAB strain was still acceptable after 2 weeks of cold storage.

## Discussion

Nowadays, products containing or processed by beneficial bacteria, such as LAB, are welcome on the market and by industry, as consumers perceive them as natural and safe. Although most beneficial LAB have been traditionally isolated from fermented dairy products, there is an increasing trend to explore novel and unconventional reservoirs of potentially helpful microbes, including both food- and nonfood-related niches [43–45]. Honey, a natural beehive food with health-promoting properties [20], is regarded as a novel source of potential probiotics for humans [21, 46]. In fresh honey, FLAB, such as *A. kunkeei* and *F. fructosus* [13, 15, 23, 24], represent the most abundant (viable) microbes*.* Even *Lactobacillus acidophilus* [26], various *Lactobacillus* spp. [47], *L. plantarum*, *Lacticaseibacillus paracasei*, *Levilactobacillus brevis*, *Lacticaseibacillus rhamnosus*, *Lacticaseibacillus casei*, *Limosilactobacillus fermentum* [24], *Lactilactobacillus curvatus* and *Pediococcus* spp. [48], *Enterococcus faecium* [27] and *Bacillus* spp. [22] were previously isolated from honey samples or other hive products.

So far, honey-isolated LAB have been characterised mainly for their antipathogenic properties [23, 48–51], with a few studies also evaluating other features that could be helpful for human applications [21, 23, 24, 46, 52].

This work addresses the isolation of LAB and FLAB strains from honey and their characterisation, in view of potential use for food biocontrol and as probiotic cultures. When selecting novel microbial candidates, different functional, technological and safety criteria need to be considered. *L. plantarum* boasts an ancient tradition of safe use for human purposes and has been already acknowledged the Qualified Presumption of Safety (QPS) status by the European Food Safety Authority [53]. Conversely, *F. fructosus*, as other FLAB, has not been evaluated yet for this attribute. However, the occurrence of FLAB in food commonly consumed by humans and the close relationship with LAB suggest that they might share similar safety features [13] and could facilitate their addition to the QPS list and the approval under the European Union ‘novel foods’ scheme [53].

Its high osmolarity and the abundance of hydrogen peroxide and other antimicrobials [54] make honey quite a harsh environment, which allows the growth of only a few well-adapted microorganisms. Particularly in mature honey, viability of LAB is greatly affected by its low water activity, so that LAB concentration ranges between 10^2^ and 10^4^ CFU g^−1^ [13]. In agreement, our honey samples contained low bacterial loads.

*Fructobacillus* spp. and *Lactobacillus* spp. isolated from honey of different floral origins are a valuable source of bacteria with antimicrobial potential [55]. In particular, honey-associated strains have been previously characterised for their ability to inhibit spoilage and clinically relevant pathogenic bacteria (i.e. *Pseudomonas aeruginosa*, *Escherichia coli*, *Bacillus subtilis*, *Staphylococcus aureus*, *Klebsiella pneumonia*) [56] and yeasts (i.e. *Candida* spp.) [48]. In this work, FLAB and LAB strains were shown to have an antifungal activity against both *B. cinerea* and *A. niger*, two moulds responsible for several harvest and post-harvest contaminations of fruits and vegetables, thus suggesting the application of the honey isolates as potential biocontrol agents [57]. Recently, Zeid et al. [58] reported that a *F. fructosus* strain, isolated from honey bee’s digestive tract, was able to antagonise *Paenibacillus larvae*, probably due to the synergistic activity of several identified bioactive compounds. In another recent study, a bacteriocin, named kunkecin A, was purified from *A. kunkeei* and shown to inhibit *Melissococcus plutonius*, the aetiological agent of European foulbrood, an important disease of honey bees [50]. In the present work, we provide a partial characterisation of the CFS compounds with antimicrobial activity, which could be attributed to organic acids, mainly lactic acid [5]. However, some differences in the rate of acidification observed between MEP3 and AREP6, and a lower inhibition detected when only lactic acid was used as negative control, indicated that further mechanisms could contribute to the antimicrobial effect. In particular, citrate, i.e. the second most abundant organic acids detected in both CFS, has been reported to increase the production of antifungal compounds in *L. plantarum* CRL 778 [59]. Similarly, succinic acid significantly inhibited the growth of foodborne pathogenic bacteria [60]. Moreover, other acids (i.e. tartaric) might participate to acidity and lowering pH or act in a synergic way with other molecules. Therefore, more studies are needed to better elucidate the role of the different compounds secreted into the medium by the investigated strains, with a particular focus on the under-explored *F. fructosus*.

Symbiotic gut FLAB are known to promote the health of honeybees [14–16]. However, to date, their probiotic potential for human application has been poorly explored [19]. Surviving stress within the host gut is mandatory for probiotics, because, even though dead microbial cells may exert health benefit as well, according to their definition, probiotics should act as live cells in the gut [28, 61]. Likewise, colonisation of the host intestinal mucosa is another relevant prerequisite of candidate probiotics, and, as recommended by WHO and FAO guidelines [62], this trait can be examined in vitro by assaying microbial adhesion to mucus and/or to cultured intestinal epithelial cells. In this study, both tested strains could survive the simulated OGI transit, with *L. plantarum* MEP3 exhibiting a higher resistance to acidic challenge which, in accordance with earlier research, represents a major stress [33, 63]. Yet, at the other digestive stages, the survival scores of MEP3 and AREP6 were similar, and overall comparable to those observed for other probiotics [33]. Recently, bee bread-isolated FLAB, i.e. *A. kunkeei*, were found to survive simulated OGI transit even better than a model probiotic strain [21]. Considering that a functional dose of probiotics should be 1 × 10^9^ CFU per serving [61, 64], the amount of culturable cells (i.e. CFU) retrieved under intestinal conditions indicates that both tested *L. plantarum* and *F. fructosus* strains could reach the colon at sufficient viable doses to initiate colonisation. Co-incubation of live bacteria with intestinal epithelial cells did not result in a cytotoxic effect. Besides, percentages of adhesion to Caco-2 monolayers were similar and in accordance with values obtained previously for *L. plantarum* isolates [45], as for other probiotic lactobacilli investigated through similar procedures [33, 65–67]. Overall, our data indicate that both tested strains could survive passage through the human gastro-intestinal tract and transiently colonise the gut.

One of the main health benefits of probiotics is the modulation of host immunity. This capacity is commonly studied through simplified in vitro systems evaluating the production of cytokines by host immune cells upon stimulation by probiotics [28]. Indeed, such approaches allow for discovering microbial strains with pro- or anti-inflammatory activities. Our findings indicate that both MEP3 and AREP6 possess immunomodulatory properties, as they were found to attenuate the transcription of TNF-α significantly in cultured macrophages, following a pro-inflammatory stimulation. Moreover, by considering the pattern of IL-10 and IL-12 expression [41, 42], AREP6 seemed to hold a greater anti-inflammatory potential, compared to MEP3. A major therapeutic objective of dietary probiotics consists in attenuating exacerbated inflammatory responses, e.g. such as those characterising inflammatory bowel diseases (IBD) [68]. Hence, both isolates could be promising for this application. While anti-inflammatory properties were previously attributed to *L. plantarum* [69–71], i.e. a species that comprises strains already present in commercialised probiotic products [3], the immunomodulatory characteristics of FLAB have been little investigated. For instance, in previous clinical pilot studies involving healthy volunteers, the dietary supplementation of heat-killed *L. kunkeei*, a FLAB obtained from honeybee products, was found to enhance IgA production [46], possibly modulate intestinal microbiota and improve bowel movement [52].

Despite a growing interest in exploiting new honey-related FLAB and LAB strains, research on their biotechnological application in the food industry is lacking. In a recent work, fructophilic *F. fructosus* and *L. plantarum* strains isolated from opuntia were used to ferment cactus pear juice and to control the growth of *Bacillus* spp. [72]. In a pioneering study, selected strictly FLAB were demonstrated to strongly reduce FODMAPs during wheat dough fermentation [73]. In this work, we have proposed the employment of honey FLAB strains with a dual purpose, i.e. as bio-protective agents and as probiotics, by using table grapes as a food model. Vegetables and fruits are considered promising foods to vehicle dietary probiotics to non-dairy consumers [74]. On the other hand, since fructophilic bacteria are natural inhabitants of fructose-rich niches, such as flowers and fruits [75], FLAB strains could also improve some functional traits of minimally processed fruits. In particular, we observed a good control over the growth of foodborne pathogenic bacterial strains, which was comparable with what was previously reported for fresh-cut pineapples and cantaloupe inoculated with *L. plantarum* or *L. fermentum* [76, 77]. Recently, the CFS from two *L. plantarum* strains were shown to delay the growth of *B. cinerea* on artificially contaminated cut kiwifruits [9]. Similarly, we found that the investigated strains were also able to inhibit the growth of filamentous fungi on the grapes. Moreover, at the end of the grape shelf life, the viability of AREP6 and MEP3 was still in the concentration range recommended to develop a probiotic effect, which is generally indicated between 10^6^ and 10^8^ CFU g^−1^ or 10^8^ and 10^10^ CFU per day [78]. We also noticed that this viability was slightly lower than what was observed previously in other fresh-cut fruits [76, 77, 79, 80]. This reduced performance to colonise the surface of fruit, which was more evident for *F. fructosus*, might depend on some structural and chemical features of grape berry cuticle, which could hinder microbial adhesion and persistence. In fact, plants are natural habitats for *L. plantarum* [81], while *F. fructosus* has been also associated to grape and wine niche [82]. Nevertheless, LAB, particularly *L. plantarum*, are more adaptable compared to FLAB, which have specialised towards a fewer fructose-rich environments. It is therefore possible that this lower flexibility might contribute to limit *F. fructosus* viability, especially under competition with other microbes. However, innovative strategies should be assessed to improve the transfer rate of probiotic bacteria on berries [83].

In conclusion, in this work, we have analysed honey LAB strains for some aspects that have been little considered before, e.g. their potential as food-protective cultures in vivo, and their probiotic character in vitro. Such kind of investigations is helpful to develop applications for humans. Interestingly, both characterised strains exhibit functional properties that encourage their use as biocontrol agents in fruit preservations and for the design of functional foods. Indeed, as these microbes derive from a fructose-rich habitat, fruit could be advantageously used both for biocontrol purposes and as a probiotic vehicle.

Additional studies, including a more comprehensive analysis of CFS, are needed to better identify the antimicrobial compounds secreted into the medium, with a particular focus on the under-explored *F. fructosus*. Moreover, our results show, in a preliminary way, that some functional properties, such as the immunomodulatory effect, seemed to be species-specific. Therefore, further investigation should be encouraged in order to elucidate the strain- or species-depending nature of the main functional features of FLAB strains isolated from different beehive sources.

## Supplementary Information

Below is the link to the electronic supplementary material.

**Fig. S1** Hyphal radial growth inhibition of *B. cinerea* CECT 20973 (**A**) and *A. niger* CECT 2805 (**B**) after three days of incubation at 24 °C in PDA plates supplemented with 10% of MRS (**a**), 10% of neutralised (**b**) or catalase-treated (**c**) CFS obtained from 48 h cultures of *L. plantarum* MEP3 or *F. fructosus* AREP6

**Fig. S2** Kinetics of bacterial growth inhibition by neutralised and catalase-treated CFS from *L. plantarum* and *F. fructosus*. *L. monocytogenes* CECT 4031 (**A**), *S. aureus* UFG141 (**B**), and *E. coli* O157:H7 UFG77 (**C**) were inoculated in TSB supplemented with 10% of MRS (circle), or with 10% of neutralised 48 h-CFS (black symbols), or 10% of 48 h-CFS treated with catalase (white symbols) obtained from *L. plantarum* MEP3 (square), or *F. fructosus* AREP6 (triangle). The cultures were incubated at 37 °C for 24 h and optical density (OD_600_) was measured at 1 h intervals. Results are the average and SD of three assays

**Fig. S3** Hyphal radial growth inhibition of *B. cinerea* CECT 20973 (**A**) and *A. niger* CECT 2805 (**B**) after three days of incubation at 24 °C in PDA plates supplemented with 10% of MRS (**a**), or 10% of MRS containing 18 g L^-1^ of lactic acid (**b**)

Supplementary file4 (PDF 358 KB)

Supplementary file5 (PDF 211 KB)

## Data Availability

The 16S rRNA sequences of the selected and investigated LAB isolates were deposited in the GenBank data library under accession numbers ON141890 (strain MEP3, *L. plantarum*) and ON141891 (strain AREP6, *F. fructosus*). The data that support the findings of this study are available from the corresponding author, upon reasonable request.
