# Supplementary figures and images for: Antimicrobial Properties, Functional Characterisation and Application of Fructobacillus fructosus and Lactiplantibacillus plantarum Isolated from Artisanal Honey
Source: Probiotics Antimicrob Proteins. 2022 Sep 29;15(5):1406–23. doi: 10.1007/s12602-022-09988-4 (PMC10491547; doi:10.1007/s12602-022-09988-4)

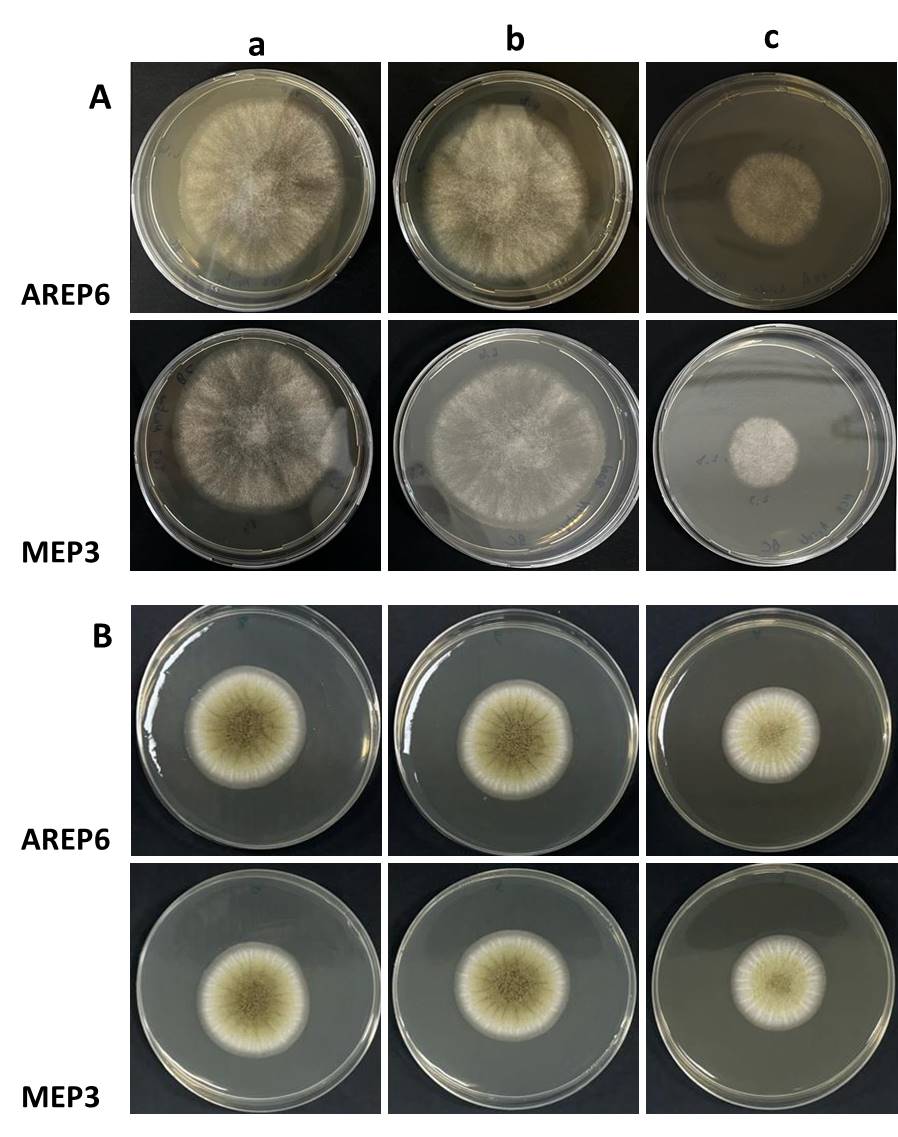

Supplement: Supplementary file 1 — Fig. S1 Hyphal radial growth inhibition of B. cinerea CECT 20973 (A) and A. niger CECT 2805 (B) after three days of incubation at 24 °C in PDA plates supplemented with 10% of MRS (a), 10% of neutralised (b) or catalase-treated (c) CFS obtained from 48 h cultures of L. plantarum MEP3 or F. fructosus AREP6 [file 12602_2022_9988_MOESM1_ESM.jpg]

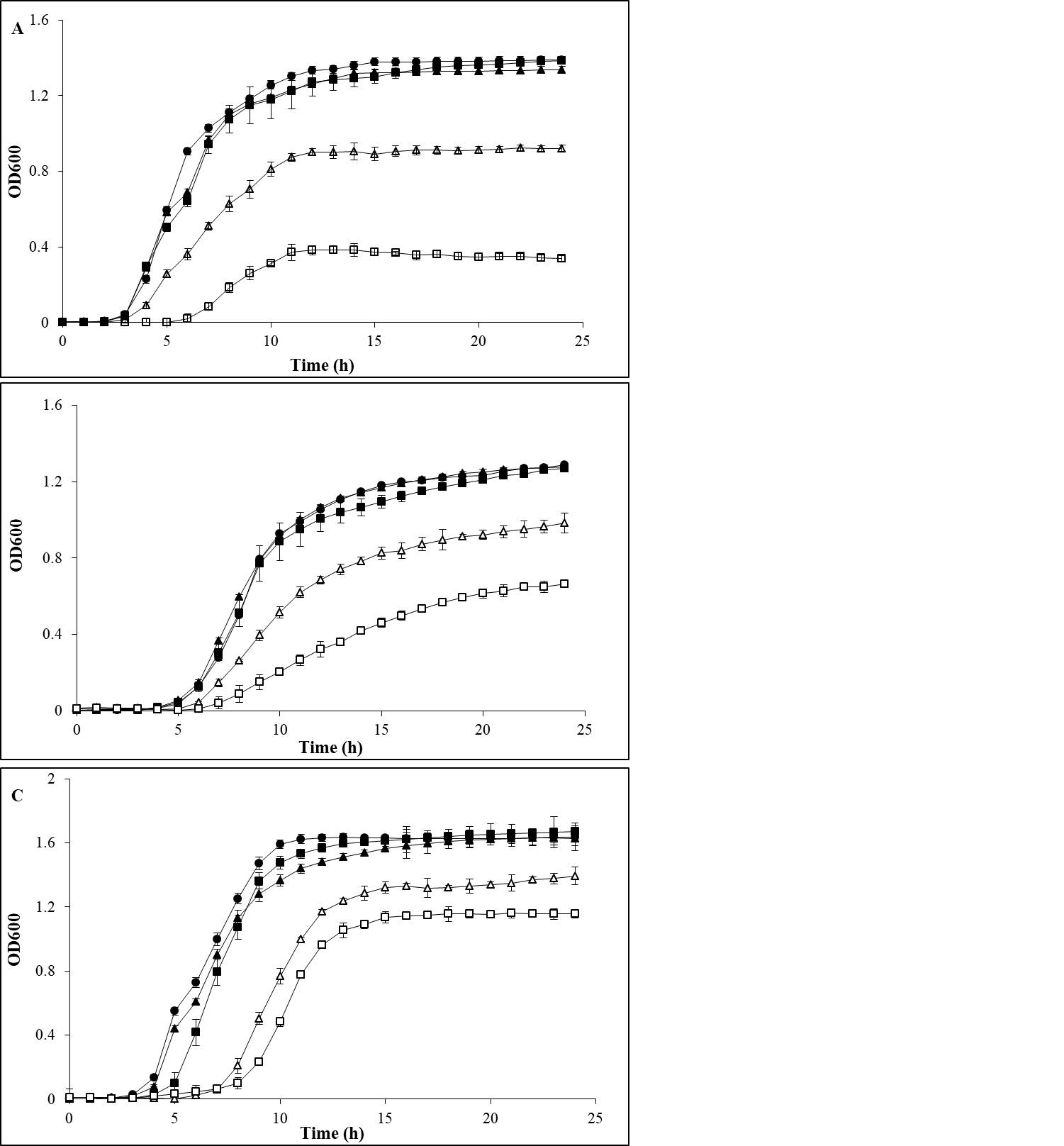

Supplement: Supplementary file 2 — Fig. S2 Kinetics of bacterial growth inhibition by neutralised and catalase-treated CFS from L. plantarum and F. fructosus. L. monocytogenes CECT 4031 (A), S. aureus UFG141 (B), and E. coli O157:H7 UFG77 (C) were inoculated in TSB supplemented with 10% of MRS (circle), or with 10% of neutralised 48 h-CFS (black symbols), or 10% of 48 h-CFS treated with catalase (white symbols) obtained from L. plantarum MEP3 (square), or F. fructosus AREP6 (triangle). The cultures were incubated at 37 °C for 24 h and optical density (OD600) was measured at 1 h intervals. Results are the average and SD of three assays [file 12602_2022_9988_MOESM2_ESM.jpg]

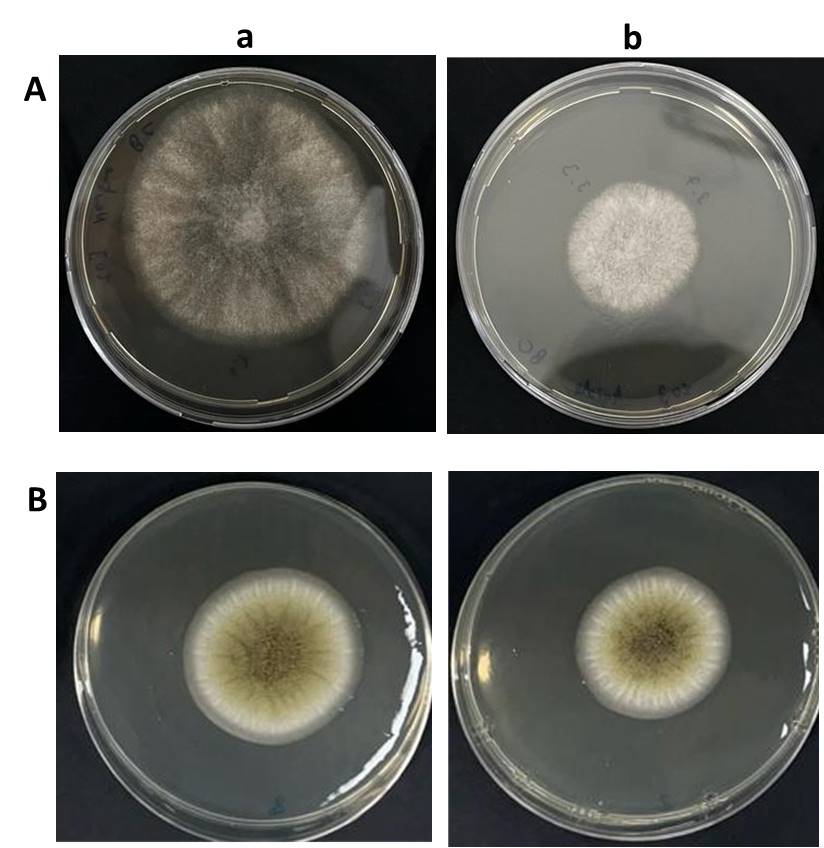

Supplement: Supplementary file 3 — Fig. S3 Hyphal radial growth inhibition of B. cinerea CECT 20973 (A) and A. niger CECT 2805 (B) after three days of incubation at 24 °C in PDA plates supplemented with 10% of MRS (a), or 10% of MRS containing 18 g L-1 of lactic acid (b) [file 12602_2022_9988_MOESM3_ESM.jpg]
