# Supplementary material for: Antimicrobial Properties, Functional Characterisation and Application of Fructobacillus fructosus and Lactiplantibacillus plantarum Isolated from Artisanal Honey
Source: Probiotics Antimicrob Proteins. 2022 Sep 29;15(5):1406–23. doi: 10.1007/s12602-022-09988-4 (PMC10491547; doi:10.1007/s12602-022-09988-4)
Supplement: Supplementary file 4 — Supplementary file4 (PDF 358 KB) [file 12602_2022_9988_MOESM4_ESM.pdf]

Table S1. Oligonucleotides used in this study

Primer name	Primer sequence (5’-3’)	Target gene, sequence ID
GAPDH F	CGACCACTTTGTCAAGCTCA	glyceraldehyde-3-phosphate dehydrogenase, NM_002046.6
GAPDH R	AGGGGTCTACATGGCAACTG	
β-act F	AAAGACCTGTACGCCAACAC	β-actin, NM_001101.4
β-act R	CATACTCCTGCTTGCTGATCC	
IL-8 F	TGTGGAGAAGTTTTTGAAGAGGG	interleukin 8, NM_000584.3
IL-8 R	CCAGGAATCTTGTATTGCATCTGG	
IL-10 F	GACTTTAAGGGTTACCTGGGTTG	interleukin 10, NM_000572.2
IL-10 R	TCACATGCGCCTTGATGTCTG	
IL-12α F	GATGGCCCTGTGCCTTAGTA	Interleukin 12α (p35), NM_001354582.1
IL-12α R	TCAAGGGAGGATTTTTGTGG	
TNF-α F	AACCTCCTCTCTGCCATCAA	tumor necrosis factor-α, NM_000594.3
TNF-α R	ATGTTCGTCCTCCTCACAGG	
