# Supplementary material for: Antimicrobial Properties, Functional Characterisation and Application of Fructobacillus fructosus and Lactiplantibacillus plantarum Isolated from Artisanal Honey
Source: Probiotics Antimicrob Proteins. 2022 Sep 29;15(5):1406–23. doi: 10.1007/s12602-022-09988-4 (PMC10491547; doi:10.1007/s12602-022-09988-4)
Supplement: Supplementary file 5 — Supplementary file5 (PDF 211 KB) [file 12602_2022_9988_MOESM5_ESM.pdf]

Table S2. Inhibition of 43 presumptive LAB isolated from different honeys against two filamentous fungi (i.e. *A. niger* CECT 2805 and *B. cinerea* CECT 20973) and three foodborne pathogenic bacteria (i.e. *E. coli* O157:H7 UFG77, *S. aureus* UFG141 and *L. monocytogenes* CECT4031) determined by the overlay assay. Isolates were classified as no (-), mild (+), or strong (++) antagonistic strains based on the size of the zone of inhibition (ZOI) around the spots. (-) halo < 1 mm; (+) 1 mm < halo <5 mm; (++) halo > 5 mm. Strains with the highest and broadest spectrum of antimicrobial activity are in bold.

**Strain**	**Honey**	***A. niger***	***B. cinerea***	***E. coli***	***S. aureus***	***L. monocytogenes***
CNP1	Coriander	-	+	++	-	+
CNP2	Coriander	-	++	-	-	++
CNP3	Coriander	-	+	+	+	-
**CNP4**	**Coriander**	++	++	++	+	++
CNP5	Coriander	+	+	+	-	+
CNP6	Coriander	+	++	+	+	+
**CNP7**	**Coriander**	+	++	++	++	+
CNP8	Coriander	-	+	+	+	+
CNP9	Coriander	+	++	+	-	-
CNP10	Coriander	-	++	+	-	+
CNP11	Coriander	-	-	-	-	-
**CNP12**	**Coriander**	+	++	++	+	++
CNP13	Coriander	-	+	+	-	-
CNP14	Coriander	-	-	+	-	+
CNP15	Coriander	+	-	++	+	+
CNP16	Coriander	-	++	+	-	-
CNP17	Coriander	-	-	+	+	-
CNP18	Coriander	-	+	++	-	+
MEP1	Wildflower	+	+	+	+	+
MEP2	Wildflower	+	+	+	-	-
**MEP3**	**Wildflower**	++	++	+	++	++
MEP4	Wildflower	-	-	++	+	+
MEP5	Wildflower	+	-	++	+	+
MEP6	Wildflower	-	+	-	-	+
MEP7	Wildflower	+	+	+	-	-
**MEP8**	**Wildflower**	+	++	++	++	+
MEP9	Wildflower	-	+	+	-	-
MEP10	Wildflower	+	++	++	+	+
MEP11	Wildflower	+	+	-	-	-
MEP12	Wildflower	-	+	+	-	+
MEP13	Wildflower	-	-	++	-	+
MEP14	Wildflower	+	-	+	-	-

MEP15	Wildflower	+	+	+	+	+
MEP16	Wildflower	-	+	+	+	+
MEP17	Wildflower	+	+	+	-	+
MEP18	Wildflower	-	+	+	-	-
AREP1	Orange	+	++	-	+	++
**AREP2**	**Orange**	**+**	**+**	**+**	**+**	**++**
AREP3	Orange	-	+	++	-	+
AREP4	Orange	+	-	+	+	++
AREP5	Orange	+	+	+	-	-
**AREP6**	**Orange**	**+**	**++**	**+**	**+**	**++**
AREP7	Orange	+	+	++	-	-
